# Commentary: Machine learning based on body composition radiomics for predicting early recurrence in colorectal cancer: a multicenter study

**DOI:** 10.3389/fnut.2026.1928055

**Published:** 2026-09-14

**Authors:** Lu Wang, Xiaohu Sun

**Affiliations:** 1 Department of General Internal Medicine, Cancer Hospital of Dalian University of Technology, Liaoning Cancer Hospital and Institute, Shenyang, China; 2 Department of Radiotherapy, Cancer Hospital of Dalian University of Technology, Liaoning Cancer Hospital and Institute, Shenyang, China

**Keywords:** body composition, colorectal cancer, decision curve analysis, early recurrence, machine learning, radiomics, sample size, scanner harmonization

Zhou et al. (1) present a multicenter study evaluating machine learning models built on body composition radiomic features from preoperative CT for predicting early recurrence in colorectal cancer. The effort to integrate nutritional status, assessed via CT-derived body composition at L3, with radiomics is commendable. Notably, the multicenter design, which includes two independent external test cohorts, represents an important strength in the radiomics literature and provides meaningful evidence of model transportability. The identification of intermuscular adipose tissue and skeletal muscle as dominant contributors in Shapley additive explanations (SHAP) analysis provides biologically relevant insights. Three methodological aspects merit discussion.

A primary concern relates to the sample size justification. The authors cite an events-per-variable (EPV) threshold, stating that with 117 events in the training cohort and 11 retained features, the resulting EPV of 10.6 satisfied this statistical requirement, referencing Riley et al. (2). Although the numerical EPV calculation is correct, two considerations warrant a more cautious interpretation. First, although 1,896 features were initially extracted, reproducibility and correlation filtering reduced the feature set to 156 before feature selection using the least absolute shrinkage and selection operator (LASSO) and Boruta; therefore, 1,896 should not be treated as the effective number of model parameters. Conversely, an EPV based only on the final 11 features does not fully capture the additional model-building complexity and potential optimism introduced by data-driven feature selection. A formal sample-size framework that accounts for outcome frequency, anticipated model performance, and model complexity—such as that described by Riley et al. (2)—would therefore provide a more defensible justification than a simple final-model EPV calculation, without implying that any single approach is the only acceptable one.

A separate but equally important issue concerns the decision curve analysis (DCA). Figure 6E of the original study demonstrates the clinical net benefit of the combined model, but the figure legend specifies “DCA in the training set”; the two external test sets, comprising 263 and 106 patients, are not represented. It should be emphasized that these external cohorts already provide meaningful evidence regarding model transportability, in the sense of predictive discrimination. What remains incompletely evaluated is specifically the generalizability of the clinical-utility claim: because net benefit depends on threshold probabilities and the clinical context in which predictions would be acted upon, favorable net benefit demonstrated outside the model-development cohort would provide additional evidence that the model could support clinical decision-making rather than merely discriminate between outcome groups. A recent colorectal cancer radiomics study similarly combined external model validation with an assessment of clinical utility (3). Reporting DCA results on both external test sets would therefore more convincingly support clinical translation.

An additional consideration involves the robustness of radiomic features to acquisition heterogeneity. Supplementary Table S1 of the original study documents six scanner configurations from three manufacturers (Siemens, Philips, and Toshiba), including a Philips IQon Spectral CT system; slice thickness ranged from 1 to 3 mm, and three reconstruction-kernel labels were used (Standard, B30f, and Fc10). Images were resampled in-plane to a pixel size of 1 × 1 mm^2^ before 2D feature extraction, and the feature matrix was subsequently standardized using Z-score normalization. While the authors suggest that body composition radiomics may be less sensitive to acquisition variability than tumor radiomics, no quantitative evidence for this assumption is provided; the extent to which the final 11 features reflect biological signal rather than technical variation therefore remains to be characterized. This is not to imply that any single harmonization algorithm is mandatory: approaches such as ComBat harmonization (4) represent one possible strategy, but their application requires careful consideration of the relationship between batch variables and biological or clinical variables, since inappropriate harmonization may remove meaningful biological information. Radiomic feature reproducibility is markedly affected by CT acquisition and reconstruction parameters (5), and the presence of two external cohorts acquired under heterogeneous conditions provides some empirical evidence of robustness, even though it does not directly quantify feature-level scanner effects. A sensitivity analysis according to vendor, reconstruction parameters, or slice thickness—or a harmonization analysis—could be presented as a possible approach to investigate this issue, rather than as a mandatory requirement.

These considerations do not diminish the value of Zhou et al.'s contribution to body composition radiomics research. A formal sample-size framework, DCA evaluation on external cohorts, and investigation of acquisition-related feature robustness should be regarded as complementary strategies for strengthening future evidence, rather than mandatory corrections to the present analysis. Incorporating them in subsequent work would further support the translation of body composition radiomic signatures into clinical practice.
